# Expert-AI Collaborative Training for Novice Endoscopists: A Path to Enhanced Efficiency

**DOI:** 10.3390/bioengineering12060582

**Published:** 2025-05-28

**Authors:** Zhen Zhang, Bai-Sheng Chen, Ling Du, Quan-Lin Li, Yan Zhu, Pei-Yao Fu, Wen-Zheng Qin, Huan-Kai Shou, Ping-Ting Gao, Xin-Yang Liu, Meng-Jiang He, Zi-Han Geng, Shuo Wang, Ping-Hong Zhou

**Affiliations:** 1 Endoscopy Center, Endoscopy Research Institute, Zhongshan Hospital, Fudan University, Shanghai 200032, China; 2 Shanghai Collaborative Innovation Center of Endoscopy, Shanghai 200032, China; 3 Endoscopic Center, Zhongshan Hospital (Xiamen), Fudan University, Xiamen 201104, China; 4 Digital Medical Research Center, School of Basic Medical Sciences, Fudan University, Shanghai 200032, China; 5 Shanghai Key Laboratory of Medical Imaging Computing and Computer Assisted Intervention, Shanghai 200032, China; 6 Data Science Institute, Imperial College, London SW7 2AZ, UK

**Keywords:** artificial intelligence, novice endoscopists, training

## Abstract

Background: Esophagogastroduodenoscopy (EGD) is essential for diagnosing upper gastrointestinal disorders. Traditional training for novice endoscopists is often inefficient and inconsistent. This study evaluates the effectiveness of an AI-assisted system (EndoAdd) in improving EGD training. Methods: In a randomized controlled trial, eight novice endoscopists were assigned to either the EndoAdd group or a control group (traditional training). The EndoAdd system provided real-time feedback on blind spots and photodocumentation. Primary outcomes were the number of blind spots, with secondary outcomes including examination time, lesion detection, and photodocumentation completeness. Results: The EndoAdd system exhibited an overall accuracy of 98.0% and a mean area under the curve (AUC) of 0.984. The EndoAdd group had significantly fewer blind spots, improved photodocumentation, and a higher lesion detection rate. Examination time was reduced without compromising diagnostic accuracy. Conclusions: The AI-assisted EndoAdd system improved novice endoscopist performance, reducing blind spots and enhancing lesion detection. AI systems like EndoAdd show potential in accelerating endoscopy training and improving procedural quality.

## 1. Introduction

Esophagogastroduodenoscopy (EGD) serves as a paramount diagnostic and therapeutic modality for upper gastrointestinal disorders, with millions of procedures performed worldwide every year. Ensuring the quality of EGD is of utmost importance to accurately diagnose and effectively manage patients’ conditions [1]. In addition to the technical and non-technical skills required for adept endoscopy manipulation, EGD training necessitates cognitive acumen and quality control measures, such as maintaining a minimal blind spot during examination [2], which profoundly influences the quality of EGD [3].

At present, it is necessary to designate an expert endoscopist as the training director in each training program. Their responsibilities include regularly monitoring trainees’ acquisition of technical and cognitive skills, maintaining comprehensive records of the trainees’ procedural experience (including indications, findings, and adverse events), assessing their performance against defined objective standards, integrating teaching resources into the program, reviewing and updating the training methodology and program quality, discussing evaluation forms with trainers and trainees, and continuously reviewing and updating the training curriculum [4]. However, in clinical practice, there has been a shortage of expert endoscopists with adequate time to supervise and train novice endoscopists.

In recent years, artificial intelligence (AI) has emerged as a transformative force across various medical disciplines, offering new possibilities to enhance clinical practice and medical education [5]. AI-assisted training systems have the potential to revolutionize EGD education by addressing the limitations of traditional training approaches [6]. The main tasks performed by AI are real-time detection, also known as computer-aided detection (CADe), and characterization, referred to as computer-aided diagnosis (CADx) [7]. Notably, AI excels at identifying blind spots during EGD procedures and reminding endoscopists to improve the quality of examination [8,9]. Additionally, this technology can enhance the completeness of photodocumentation. Inspired by these applications, we believe that AI systems can serve as training directors during novice EGD training [10], helping inexperienced endoscopists improve their gastroscopy operations while avoiding blind spots. If effectively applied, this system could significantly alleviate the training burden on expert endoscopists and enable the training of more qualified endoscopists to meet the demands of gastrointestinal endoscopy [11], thus improving the access to high-quality endoscopic services, ensuring accurate diagnoses, and optimizing patient care [11].

Despite existing AI tools for quality control in endoscopy, there remains a lack of systems specifically designed to support novice training in a structured and real-time manner. Novice endoscopists often struggle with recognizing anatomical landmarks and ensuring complete mucosal inspection without expert supervision. This not only leads to variability in training outcomes but also increases the risk of blind spots and missed lesions. Prior study have demonstrated that AI-based systems can significantly improve novice trainees’ performance in esophagogastroduodenoscopy (EGD), particularly by reducing blind spots and enhancing mucosal visualization [4]. Similarly, systematic reviews have established the value of virtual reality (VR) simulators in accelerating the acquisition of technical skills and reducing patient discomfort, although these technologies often lack real-time AI guidance and are commonly limited to simulated settings [12,13]. Moreover, international position statements and multi-center surveys, including those by the European Society of Gastrointestinal Endoscopy (ESGE), have emphasized the importance of standardized curricula, simulation-based learning, and competency-based assessment in endoscopy training [14,15]. However, most prior AI-focused studies have been limited to single centers, single phases, or simulated environments, and have not integrated real-time AI guidance, structured feedback, and longitudinal assessment within real-world, multi-phase clinical training [4,12,16,17].

Recognizing these challenges, we were motivated to develop an AI-based assistant that could serve as a real-time training director, offering immediate feedback on anatomical coverage, blind spot detection, and photodocumentation completeness. Such a system aims to enhance procedural learning, reduce reliance on expert trainers, and promote standardization in endoscopy education.

## 2. Materials and Methods

### 2.1. Development of the EndoAdd Teaching System

**Figure 1:**
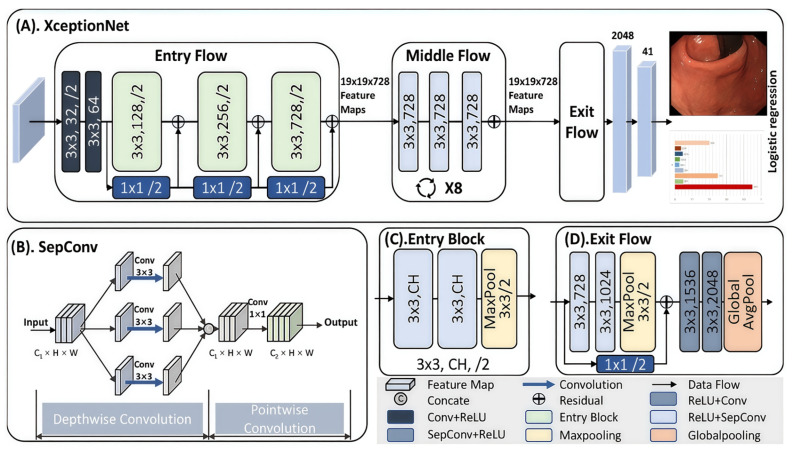
The system design of EndoAdd system. (**A**) The architecture of XceptionNet and its prediction. Specifically, the EGD image is classified into 26 predefined classifications. (**B**) Illustration of the SepConv representing the depthwise separable convolution used in the XceptionNet. (**C**) The entry block representing the basic block of entry flow in XceptionNet. (**D**) The module illustration of exit flow of XceptionNet. The notation for the convolutional module is k × k, channel/skip, where k represents the convolution kernel size, channel represents the feature channel, and skip represents the skip of the convolution kernel.

To aid novice trainees, a deep learning model based on a convolutional neural network (CNN) was developed to classify EGD images into 26 predefined categories representing upper gastrointestinal tract sites. The goal was to assist in their training, as depicted in Figure 1.

### 2.2. Dataset

A dataset comprising EGD images from 5000 patients was constructed to train and validate the EndoAdd system. In vitro images were selected by a junior endoscopist for further annotation. Two senior endoscopists independently labeled these images into 27 categories, including 26 different upper gastrointestinal tract sites and “NA” (not applicable). Another senior endoscopist reviewed the images and labels to ensure quality control and resolve any disagreements between the two endoscopists. Images from 500 patients were randomly divided into training (80%, 400 patients, 35,974 images), validation (10%, 50 patients, 5415 images), and test (10%, 50 patients, 5564 images) sets at the patient level.

### 2.3. Network Architecture

**Figure 2:**
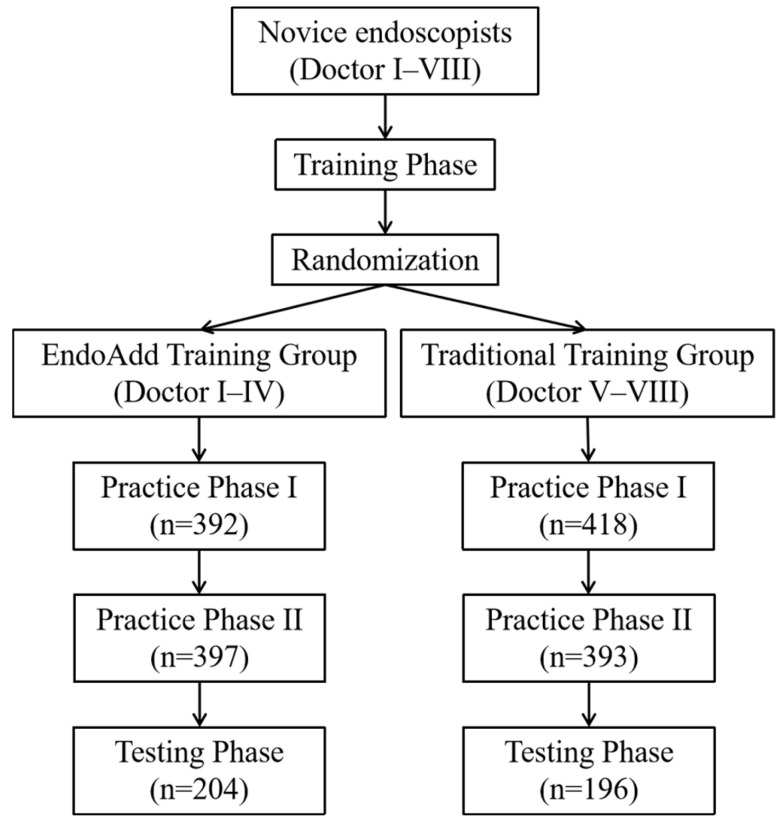
Flow diagram.

The EndoAdd system employed XceptionNet [11] as the backbone network for EGD frame classification. This convolutional neural network was selected for its efficiency and strong performance in medical image recognition tasks. As shown in Figure 1A, the architecture consists of three main parts: (1) the entry flow, which extracts low-level features such as edges and contours from input images (Figure 1C); (2) the middle flow, repeated eight times, which uses depthwise separable convolutions to extract higher-level semantic features; and (3) the exit flow, which fuses features through global average pooling and a fully connected layer to generate final predictions (Figure 1D). The core building block is the depthwise separable convolution module (Figure 1B), an enhanced version of the Inception module [18], designed to reduce the number of learnable parameters while maintaining feature extraction capability. In the EndoAdd system, each incoming EGD video frame is resized and preprocessed before being fed into the XceptionNet model. The model outputs a probability distribution across 26 predefined anatomical classes and one “NA” (not applicable) class. These outputs are used to determine the current anatomical region being visualized in real time. The classification results are continuously logged and aggregated to track which anatomical sites have been inspected. Based on this, the system dynamically generates a real-time blind spot map and provides both live procedural guidance and post-procedure summaries. The entire classification system was implemented using PyTorch 1.8 and deployed on a workstation configured for real-time inference (~25 fps), enabling seamless integration into the clinical endoscopy workflow. This classification component forms the core of EndoAdd’s functionality in training support and procedural quality assurance.

### 2.4. Network Training

EGD images were inputted into the network and the outputs were probabilities corresponding to 26 different upper gastrointestinal tract sites and “NA”. The network weights pretrained on ImageNet [19] were adapted and fine-tuned using our in-house EGD dataset. The binary cross-entropy loss function was utilized:

(1) $$\mathrm{Loss}=-\frac{1}{N}\sum_{i}^{N}\sum_{j}^{M}y_{ij}\log\left(p_{ij}\right),$$

where N is the number of samples, M is the number of classes, p_ij_ is the predicted probability, and y_ij_ is the ground-truth label annotated by endoscopists. The neural network was implemented using PyTorch 1.8 on a workstation with an Intel Core i7-6700K CPU, 32 GB RAM, and NVIDIA GTX1060 GPU with 6 GB memory.

#### 2.4.1. Data Augmentation

To enhance model generalizability and reduce the risk of overfitting, we implemented comprehensive data augmentation strategies during training. These included random rotations within ±15°, horizontal and vertical flipping, scaling between 95% and 105%, random cropping, and color jitter (adjustment of brightness and contrast within ±10%). Each augmentation was applied with a defined probability to each training image, ensuring a diverse training dataset.

#### 2.4.2. Hyperparameter Optimization

Model hyperparameters—including learning rate, batch size, number of epochs, and dropout rate—were optimized using a grid search approach on the training dataset. The grid search covered learning rates from 1 × 10^−5^ to 1 × 10^−3^, batch sizes of 16 and 32, and dropout rates ranging from 0.2 to 0.5. The final hyperparameter set was selected based on the highest performance metrics obtained on the validation set during cross-validation.

#### 2.4.3. Model Validation

Model performance and stability were assessed using stratified five-fold cross-validation within the training set. Hyperparameter tuning was conducted based on validation results in each fold. For final evaluation, the model was retrained on the combined training and validation data using the optimized hyperparameters, and tested on the independent held-out test set. Performance metrics, including accuracy, sensitivity, specificity, and the area under the receiver operating characteristic curve (AUC), were calculated to comprehensively assess the model’s effectiveness. An early stopping strategy was applied based on validation loss to prevent overfitting. The final model was evaluated on the test set and performance was reported as accuracy and AUC.

### 2.5. Trial Design

This study was designed as a prospective, randomized controlled trial. The study consisted of three phases: the training phase, the practicing phase, as well as the test phase. The entire study protocol was approved by the institutional review board of Zhongshan Hospital (B2021-805R). It was registered at the Chinese Clinical Trial Registry (ChiCTR2200062730). All authors had access to the study data and reviewed and approved the final manuscript.

Eight novice trainees without prior EGD operational experience were recruited for this research between 8 August 2022 and 31 January 2023. The flow chart of the study is depicted in Figure 2. All endoscopic images used for model development and clinical procedures were obtained using the Olympus GIF-H290 HD endoscope system (Olympus Medical Systems, Tokyo, Japan) under standard white-light imaging mode.

### 2.6. Training Phase

Before performing EGD procedures, the eight novice trainees completed a comprehensive training course. The course covered basic concepts of indications and contraindications for EGD, the process of EGD, the operating demonstrations, the introduction to common endoscopic instruments, as well as the diagnosis of upper gastrointestinal diseases. The training course lasted approximately 10 h and involved five experienced endoscopists and one nurse as instructors. Subsequently, the trainees were allowed to observe EGD procedures in the examination room and perform five EGDs with the assistance of senior endoscopists. Before concluding the training phase, the trainees were required to take an exam consisting of twenty multiple-choice and five short-answer questions assessing their knowledge of basic EGD concepts, anatomical structures, and common lesions.

### 2.7. Randomization and Blinding Procedure of the Practicing and Testing Phase

Before commencing the practicing and testing phase, the eight novice trainees selected grouped envelopes and were randomly assigned to either the EndoAdd group or the control group, with each group consisting of four trainees.

During outpatient visits when scheduling EGD appointments, patients were interviewed by a research assistant. Written informed consent was obtained from all patients. Meanwhile, the assistant explained the aims of this study and collected demographic and medical information using a data collection sheet. Eligible participants were randomized into the EndoAdd group and the control group in a 1:1 ratio through block randomization with stratification by center. The random allocation table was generated using SAS 9.4 software, and the masking of randomization was facilitated with opaque envelopes. Patients remained blinded to their group assignment.

To minimize performance and assessment bias, senior trainers who supervised the EGD procedures were not informed of the group assignments (EndoAdd or control) of the novice trainees during the testing phase. During this phase, the EndoAdd system did not display real-time feedback, ensuring that both groups underwent procedures under identical visual and procedural conditions. Furthermore, all procedural recordings were anonymized and coded before being reviewed by independent assessors who were blinded to group allocation. During the practicing phases, while it was necessary for trainers to be aware of the intervention due to the visible presence of the EndoAdd system in the AI group, direct trainer input was restricted by protocol. Trainers were instructed not to intervene or provide feedback during the examination unless patient safety was at risk. Their role was limited to observation and emergency intervention, thereby reducing the risk of introducing bias based on group knowledge.

### 2.8. Patient Recruitment of the Practicing and Testing Phase

A cohort of outpatients aged between 18 and 75 years who were scheduled to undergo routine diagnostic EGD were enrolled in this study. Participants were required to willingly provide informed consent. Additionally, specific exclusion criteria were implemented to maintain the study’s internal validity. These exclusion criteria encompassed the following conditions: (1) history of prior surgical interventions related to esophageal, gastric, duodenal, small intestinal, or colorectal cancer; (2) presence of gastroparesis or gastric outlet obstruction; (3) severe chronic renal failure, defined as a creatinine clearance below 30 mL/min; (4) severe congestive heart failure, classified as New York Heart Association class III or IV; (5) ongoing pregnancy or breastfeeding; (6) diagnosis of toxic colitis or megacolon; (7) poorly controlled hypertension, indicated by systolic blood pressure exceeding 180 mm Hg and/or diastolic blood pressure surpassing 100 mm Hg; (9) moderate or substantial active gastrointestinal bleeding, quantified as greater than 100 mL per day; and (10) existence of major psychiatric illness.

### 2.9. Interventions of the Practicing and Testing Phase

Patients were randomly assigned to either the normal group or the EndoAdd group and all EGD examinations took place between 8:30 and 11:30 or 13:30 and 16:30. All EGD procedures in this study were performed under deep sedation using intravenous propofol, administered by anesthesiologists according to institutional protocols, to ensure consistent baseline conditions across all participants. In addition, a scopolamine butylbromide was routinely administered prior to the examination unless contraindicated. The examination was supervised by senior trainers with over 5000 EGD experiences.

During the practicing period, doctors in the EndoAdd group performed an examination with the assistance of the EndoAdd system; the normal group performed an examination with the assistance of senior doctors. During the testing period, doctors in both groups performed an examination under the surveillance of senior doctors, without the assistance of the EndoAdd system. Real-time blind spots were displayed on the screen and novice trainees in this group could refer to this information during the EGD procedure. If any EGD lesions were detected, trainees were allowed to perform biopsies before concluding the procedure. Once they confirmed the completion of the EGD, the screen displayed their performance, including remaining blind spots, the procedure route, and photodocumentation recorded from the EGD. Senior trainers were not permitted to provide additional information.

Patients in the normal group were examined by the novice trainees using a routine inspection process. No additional information was shown on the screen. If any EGD lesions were detected, trainees were allowed to perform biopsies before concluding the procedure. Trainers could provide instructions on EGD procedures.

The time allotted for the EGD by novice trainees was limited to 10 min, excluding biopsy time, to ensure patient well-being. Senior trainers had the authority to stop the EGD procedure if they anticipated adverse events. All patients underwent a repeat EGD performed by a senior trainer to prevent missed diagnoses.

### 2.10. Outcome

The primary outcome of the study was the number of blind spots in the control and EndoAdd groups. The secondary outcomes included: (1) blind spot rate (number of unobserved sites in each patient/26 × 100%); (2) inspection time; (3) detection rate of the lesions; and (4) completeness of photodocumentation produced by endoscopists.

### 2.11. Statistical Analysis and Sample Size Calculation

The number of blind spots observed among trainees at our endoscopic centers was approximately 5.8. We hypothesized that the app would increase the number of blind spots to 2. To detect this difference accompanied with a significance level (α) of 0.05 as well as a power of 80% based on a two-tailed test, the sample size for this study was calculated to be approximately 322 patients. Considering that approximately 20% of patients may cancel their colonoscopy appointments, we estimated that a total of 400 patients would be necessary for the discovery of a statistically significant difference in the primary outcomes.

All statistical analyses were performed using SAS software (version 9.4). To address the risk of inflated type I error due to multiple comparisons across anatomical regions and time points, we applied the Benjamini–Hochberg procedure to control the false discovery rate (FDR) for all relevant outcome measures. Adjusted *p*-values were reported and a two-tailed *p*-value of <0.05 after correction was considered statistically significant. As secondary outcomes were considered exploratory, statistical multiplicity resulting from multiple outcomes was not corrected in this study.

Continuous variables were presented as mean ± standard deviation (SD) and compared using student’s *t*-test. Categorical variables were presented as numbers (percentages) and analyzed using either the chi-square test or Fisher exact test. To estimate the rate and its 95% confidence interval (CI) for each group, we employed the Clopper–Pearson method. Additionally, we calculated the rate difference between the two groups and its 95% CI using the Newcombe–Wilson method with a continuity correction. In certain subgroups, we also compared the rate of adequate bowel preparation between the study group and the control group.

## 3. Results

### 3.1. Performance of the EndoAdd System on Image Classification

**Figure 3:**
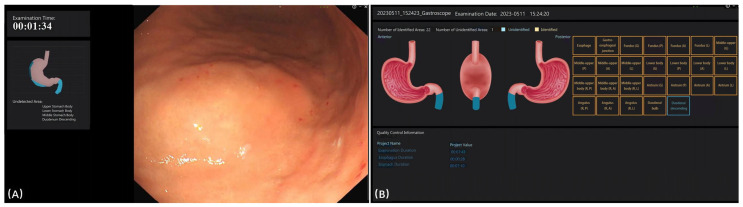
(**A**) The interface of the EndoAdd system during examination. The blind spot is shown in the left part on the screen. (**B**) The interface of the EndoAdd system after examination.

Figure 3 displays the interface of the EndoAdd system during and after examination. During the examination, the blind spot was presented on the left side of the screen (Figure 3A). After the examination, any missed categories were displayed on the screen (Figure 3B). The EndoAdd system demonstrated robust performance in EGD image classification. After approximately 300 training epochs, the model achieved an overall accuracy of 98.0% and a mean area under the curve (AUC) of 0.984 on the test set. The system provided real-time feedback during examinations by highlighting blind spots and, after each procedure, displayed any missed anatomical sites (Figure 3). Detailed performance metrics for each anatomical category are available in Supplementary Table S1.

### 3.2. Trainee Characteristics and Knowledge Assessment

After completing the training phase, the eight trainees took an exam consisting of twenty multiple-choice questions and five short-answer questions designed to assess their knowledge of basic concepts, anatomical structures, and common lesions related to EGD. The two groups of trainees demonstrated comparable understanding and performance levels (25.5 ± 2.38 vs. 26 ± 2.16, *p* = 0.31) regarding the material covered during the training phase (Supplementary Tables S2 and S3). However, it is important to note that this exam primarily assessed theoretical knowledge and its results may not directly correlate with practical performance.

### 3.3. Post-Training Outcomes

Baseline information of patients is shown in Table 1. The comparison of pre- and post-training outcomes for the eight endoscopists (see Supplementary Tables S2 and S3) revealed a statistically significant decrease in average examination time in both the EndoAdd group and traditional training group for most endoscopists (*p* < 0.01), including Doctors I, II, III, IV, VII, and VIII. Additionally, a statistically significant reduction in blind spots was observed across all endoscopists (*p* < 0.01). Most endoscopists also demonstrated improvements in the completeness of photodocumentation (*p* < 0.01).

The EndoAdd group exhibited significant reductions in omission rates across various anatomical areas, including the Middle-upper, Middle-upper body, Angulus, as well as selected areas of the Antrum and Fundus. These improvements ranged from 4 to 10 significant improvements per physician, totaling 28 improvements. In contrast, the traditional training group showed fewer significant improvements, ranging from 3 to 7 improvements per physician, totaling 19 improvements, which were primarily concentrated in the Middle-upper and Middle-upper body areas.

### 3.4. Analysis of the Practicing Phases and Testing Phase

**Figure 4:**
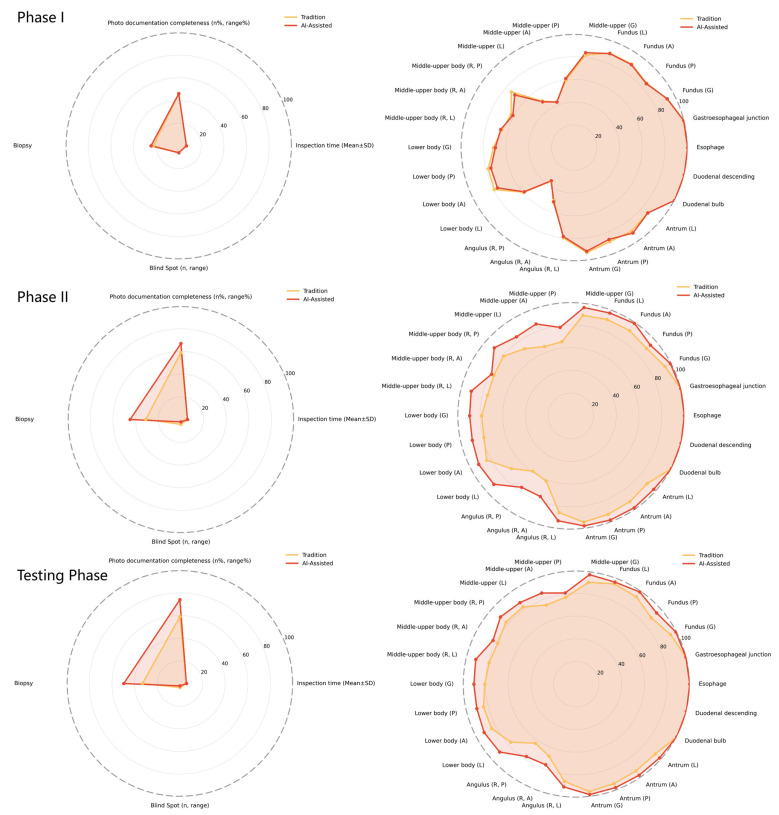
Overall performance indicators during practice phase I, practice phase II, and the testing phase for the EndoAdd and the traditional training group.

Further analysis was conducted across three distinct phases: practicing phase I, practicing phase II, and the testing phase. Table 2 and Figure 4 present a comprehensive comparison of outcomes for both the EndoAdd and traditional training groups. Prior to training, no statistically significant differences were observed between the EndoAdd group and the traditional training group regarding examination time, missed diagnosis rates in various anatomical areas, or biopsy rates.

During practicing phase II, the EndoAdd group demonstrated superior performance across multiple indicators compared to the traditional training group. Notable improvements included enhanced completeness of photodocumentation (67 (45, 88) vs. 59 (30, 79), *p* < 0.01), higher biopsy rates (44.84% vs. 31.04%, *p* < 0.01), and reduced omission rates in specific regions such as the Fundus, Middle-upper, Middle-upper body, Lower body, Angulus, and selected areas of the Antrum (*p* < 0.01). Similar trends persisted during the testing phase.

Notably, during practicing phase II, the EndoAdd group achieved a satisfactory level of examination proficiency comparable to that of experienced endoscopists. In the subsequent testing phase, the AI-assisted group demonstrated significant advantages, including improved photodocumentation completeness (74 (18, 92) vs. 59 (28, 82), *p* < 0.01).

### 3.5. Diagnostic Rates and Lesion Detection

Regarding the diagnostic rates and lesion detection, an evaluation was conducted to compare the performance of both the EndoAdd group and the traditional training group in diagnosing benign and malignant lesions. The experienced endoscopists were considered as the gold standard for this assessment. The findings indicated that both groups achieved diagnostic rates comparable to those of the experienced endoscopists in identifying conditions such as H. pylori infection, ulcers, polyps, submucosal elevations, and early-stage gastrointestinal cancers. Notably, there were no significant differences observed in the lesion detection rates between the two groups, as depicted in Table 3.

## 4. Discussion

The growing demand for EGD services amidst a shortage of skilled endoscopists underscores the limitations of traditional training methods, which are often inefficient, variably safe, and dependent on individual trainers’ expertise [20,21,22]. Emerging evidence demonstrates that AI-assisted training can achieve diagnostic accuracy comparable to experienced endoscopists [20,21], suggesting its potential to address these challenges. This study systematically evaluated the efficacy of EndoAdd, an AI-assisted training system, compared to conventional methods in novice endoscopist education. Both training approaches improved endoscopist performance across multiple metrics, but EndoAdd demonstrated distinct advantages. Notably, AI guidance reduced blind spots more effectively than traditional training (*p* < 0.05). The real-time feedback mechanism likely enhanced lesion detection by improving anatomical awareness and procedural consistency, particularly in complex regions such as the Angulus, partial Antrum, and Fundus [23]. These findings align with prior reports on AI’s capacity to standardize gastrointestinal endoscopy practices [22]. By bridging the gap between algorithmic development and practical, standardized deployment of AI in endoscopy education, our study contributes new evidence and a practical framework for future research and clinical adoption of AI-guided training.

Photodocumentation completeness—a critical factor for accurate diagnosis and interdisciplinary communication improved significantly in both groups, with EndoAdd showing superior enhancement (*p* < 0.01). The system’s automated imaging guidance ensured comprehensive gastrointestinal tract visualization, facilitating precise documentation without compromising procedural efficiency. Both groups achieved comparable reductions in examination time, indicating that AI integration does not impede workflow dynamics. During phase II training, EndoAdd trainees attained proficiency levels equivalent to experienced endoscopists in both blind spot reduction and photodocumentation (*p* < 0.05), whereas traditional trainees demonstrated slower progression. This accelerated skill acquisition translated to fewer required training cases for AI-assisted learners (median 28 vs. 41 cases; *p* = 0.003). Importantly, neither method compromised diagnostic accuracy for common lesions (*p* > 0.05), confirming AI training’s non-inferiority in developing core competencies. Qualitative assessments corroborated quantitative findings: EndoAdd users reported heightened confidence in independent procedure execution due to real-time blind spot visualization, while traditional trainees emphasized the value of mentorship despite challenges in self-directed error correction [21]. These observations suggest that AI systems may optimally function as adjuncts to human supervision, providing standardized, objective feedback to complement experiential learning.

While a formal cost-effectiveness analysis was beyond the scope of this study, initial considerations suggest that implementing the EndoAdd system would involve costs related to hardware, software licensing, and user training. However, potential benefits may include increased training efficiency, reduced demand for direct expert supervision, and shorter learning curves, which could translate into long-term cost savings for training programs.

Economic modeling indicates that EndoAdd implementation could yield long-term cost efficiencies through reduced expert supervision requirements and increased procedural throughput, despite initial infrastructure investments. However, successful clinical integration requires addressing technical compatibility with existing endoscopic platforms, workflow optimization, and regulatory compliance—including GDPR-compliant data handling protocols. Prospective validation under formal regulatory frameworks remains essential to ensure safety and accountability in AI-driven medical education.

The integration of artificial intelligence (AI) into medical training also introduces several important ethical considerations that must be thoughtfully addressed to ensure safe, effective, and equitable practice. While AI systems offer valuable real-time feedback and standardized guidance, there is a risk that over-reliance may undermine the development of independent decision-making and critical thinking in trainees, making it essential for training programs to foster core competencies and professional judgment with AI serving as a supportive tool rather than a replacement for human expertise. Additionally, the use of AI-assisted systems raises important questions regarding patient consent, as patients should be fully informed when AI is involved in their care, including the potential benefits, limitations, and data privacy considerations, to maintain transparency and trust. The introduction of AI may also affect the doctor-patient relationship, as technology can enhance procedural quality and patient safety, but may also lead to concerns about depersonalization if not carefully managed. Therefore, open communication and a commitment to maintaining empathy and interpersonal connection are critical to ensuring that AI enhances rather than diminishes the human aspect of care. Overall, the responsible adoption of AI in endoscopy training depends on proactive attention to these ethical issues, with ongoing dialogue among educators, clinicians, patients, and ethicists guiding the integration of technology into medical education.

This study demonstrates that AI-assisted training effectively addresses key limitations of traditional endoscopy education while maintaining diagnostic rigor. Future research should investigate longitudinal skill retention and broader implementation strategies across diverse healthcare settings.

## 5. Limitations

The EndoAdd system demonstrates notable advantages in minimizing blind spots and improving photodocumentation quality; however, several critical limitations require consideration. Firstly, the training dataset’s single-center origin and East Asian predominance may compromise external validity across ethnically diverse populations and clinical settings with differing disease prevalence patterns. While encompassing common pathologies, the system’s diagnostic performance metrics approached ceiling levels in both intervention and control groups for many lesion types. This ceiling effect likely attenuated our ability to detect statistically meaningful differences attributable to AI assistance, potentially underestimating the intervention’s incremental value. These findings underscore the necessity for human oversight in clinical deployment, particularly for rare or complex lesions. Secondly, the pilot study’s limited sample size constrains generalizability. The small cohort fails to capture inter-individual variability in baseline competencies and learning trajectories, while the visible presence of the EndoAdd system rendered complete blinding of trainers during practice phases unfeasible. This potential source of performance bias may have influenced trainee behavior or supervision intensity despite protocol restrictions on direct intervention. Additionally, the absence of longitudinal follow-up precludes assessment of skill retention and autonomous application post-AI training. Thirdly, the lack of formal cost-benefit analysis warrants attention. Although potential long-term gains in procedural quality exist, substantial upfront implementation costs may hinder adoption in resource-constrained settings. Fourthly, informal qualitative feedback was collected from trainees after the intervention. Key themes included increased confidence in anatomical recognition, perceived usefulness of real-time guidance (AI group), and suggestions for more interactive features. However, this feedback was anecdotal and not obtained through a structured questionnaire. Future studies should incorporate standardized, quantitative measures to more rigorously capture trainee perspectives. Fifth, AI integration in early training raises concerns regarding overdependence effects. Prolonged reliance on automated guidance might attenuate the development of critical competencies including independent clinical judgment, adaptive decision-making, and pattern recognition skills. Future technical refinements should prioritize enhanced lesion characterization algorithms, real-time procedural guidance, and optimized user interface design. Addressing real-world implementation challenges requires standardized interoperability protocols, reduced computational latency under clinical workload pressures, and seamless integration with heterogeneous hospital IT infrastructures. Particular attention must be given to mitigating cognitive burden on endoscopists through streamlined workflow integration. To address the ceiling effect limitation, future studies should incorporate more challenging or subtle lesion cases (e.g., flat neoplasms, early cancers) and employ granular assessment methodologies such as confidence scoring, lesion conspicuity metrics, or eye-tracking analysis to better quantify diagnostic improvements. Multicenter trials with extended follow-up periods are essential to validate the system’s scalability, cost-effectiveness, and educational sustainability. Complementary developments should focus on multimodal data fusion strategies, algorithmic improvements in detection specificity, and bidirectional human-AI interaction frameworks to enhance training efficacy.

## 6. Conclusions

In conclusion, the findings of this study underscored the potential benefits of AI-assisted training in improving the performance of novice endoscopists. The EndoAdd group exhibited superior outcomes in terms of reduced examination time, decreased blind spots, improved the completeness of photodocumentation, and enhanced detection rates in specific anatomical areas. These results support the notion that AI technology can be a valuable tool in endoscopy training, facilitating skill development and enhancing overall endoscopist proficiency. Further research is necessary to delve into the long-term impact, cost-effectiveness, and generalizability of AI-assisted training across various clinical settings and skill levels. The integration of AI technology into the training programs has the potential to advance the field of endoscopy and improve patient outcomes.

## Figures and Tables

**Table 1:** Baseline characteristics for patients who underwent esophagogastroduodenoscopy.

	Practice Phase I (n%)		Practice Phase II (n%)		Testing Phase (n%)	
	AI (n = 392)	Tradition (n = 418)	AI (n = 397)	Tradition (n = 393)	AI (n = 204)	Tradition (n = 196)
Age (Mean ± SD)	53.07 ± 18.17	51.08 ± 18.13	51.37 ± 17.92	52.72 ± 18.11	55.65 ± 17.70	55.07 ± 18.17
Gender (F, n%)	49.5	48.8	45.3	46.6	52.9	49.5
Abdominal discomfort	33.2	30.6	31.0	34.1	31.9	30.1
Acid reflux	9.7	10.5	10.3	7.9	10.3	12.8
Anaemia	1.5	1.7	1.0	1.3	2.0	1.5
Belching	3.3	3.1	2.8	2.8	2.5	4.1
Bowel habit change	1.8	2.9	2.0	2.3	2.5	3.6
Constipation	3.1	2.4	2.3	2.5	3.9	3.6
Dyspepsia	2.8	3.3	3.5	2.5	3.4	2.6
Dysphagia	3.1	3.1	2.8	3.8	2.0	4.1
Emaciation	1.0	1.0	0.5	1.0	1.0	0.5
Health examination	19.4	18.4	19.9	18.1	17.2	16.3
Poor appetites	16.8	17.0	18.9	18.6	19.6	17.9
Suspected GI bleeding	1.0	2.2	1.5	1.8	1.0	1.0
Suspected malignancy	1.3	1.4	1.0	1.5	1.0	1.0
Vomiting	2.0	2.4	2.5	1.8	2.0	1.0

**Table 2:** Overall performance indicators during practice phase I, practice phase II, and the testing phase for the EndoAdd and the traditional training group.

	Practice Phase I (n%)				Practice Phase II (n%)				Testing Phase (n%)			
	AI-Assisted	Tradition	*p*-Value	FDR	AI-Assisted	Tradition	*p*-Value	FDR	AI-Assisted	Tradition	*p*-Value	FDR
	(n = 392)	(n = 418)			(n = 397)	(n = 393)			(n = 204)	(n = 196)		
Inspection time (Mean ± SD)	6.81 ± 1.44	6.85 ± 1.49	0.696	0.878	5.81 ± 1.25	5.89 ± 1.28	0.349	0.498	5.76 ± 1.60	5.86 ± 1.51	0.542	0.711
Photo documentation completeness (n%, range%)	46 (13, 82)	46 (18, 86)	0.788	0.878	67 (45, 88)	59 (30, 79)	*p* < 0.01	0.013 *	74 (18, 92)	59 (28, 82)	*p* < 0.01	0.014 *
Biopsy	24.49	22.49	0.502	0.878	44.84	31.04	*p* < 0.01	0.013 *	49.59	33.08	*p* < 0.01	0.014 *
Blind spot (n, range)	6.09 ± 2.55	5.97 ± 2.56	0.485	0.878	2.10 ± 1.15	4.27 ± 1.43	*p* < 0.01	0.013 *	2.00 ± 2.88	3.48 ± 2.30	*p* < 0.01	0.014 *
Esophage	0.00	0.00	1.000	1	0.00	0.00	1.000	1	0.00	0.00	1.000	1
Gastroesophageal junction	0.51	0.72	0.706	0.878	0.25	0.25	0.994	1	0.49	0.51	0.750	0.789
Fundus (G)	7.40	7.66	0.89	0.89	1.01	6.36	*p* < 0.01	0.013 *	0.98	6.12	*p* < 0.01	0.014 *
Fundus (P)	15.05	14.59	0.855	0.878	6.30	10.69	0.027	0.087	5.39	11.73	0.040	0.045 *
Fundus (A)	11.22	10.77	0.835	0.878	1.26	8.91	*p* < 0.01	0.013 *	1.47	6.63	*p* < 0.01	0.014 *
Fundus (L)	11.48	11.00	0.831	0.878	3.27	9.41	*p* < 0.01	0.013 *	3.92	5.61	0.030	0.045 *
Middle-upper (G)	15.82	17.94	0.420	0.725	4.03	10.94	*p* < 0.01	0.013 *	2.94	9.69	*p* < 0.01	0.014 *
Middle-upper (P)	38.78	40.19	0.681	0.878	21.66	34.35	*p* < 0.01	0.013 *	19.12	22.96	*p* < 0.01	0.014 *
Middle-upper (A)	57.14	56.94	0.953	0.953	13.60	34.86	*p* < 0.01	0.013 *	14.22	25.51	*p* < 0.01	0.014 *
Middle-upper (L)	51.02	50.24	0.824	0.878	15.62	28.24	*p* < 0.01	0.013 *	12.75	17.35	*p* < 0.01	0.014 *
Middle-upper body (R, P)	30.10	26.08	0.203	0.725	9.82	20.87	*p* < 0.01	0.013 *	10.78	17.35	0.020	0.029 *
Middle-upper body (R, A)	39.03	37.32	0.617	0.878	21.16	23.41	0.447	0.633	17.16	21.94	0.450	0.616
Middle-upper body (R, L)	33.42	34.45	0.757	0.878	9.57	24.43	*p* < 0.01	0.013 *	8.82	20.92	*p* < 0.01	0.014 *
Lower body (G)	30.61	28.95	0.605	0.878	10.83	21.37	*p* < 0.01	0.013 *	9.80	19.39	*p* < 0.01	0.014 *
Lower body (P)	24.49	22.01	0.404	0.725	10.58	21.12	*p* < 0.01	0.013 *	9.80	15.82	0.010	0.029 *
Lower body (A)	23.72	20.57	0.280	0.725	8.31	16.28	*p* < 0.01	0.025 *	8.33	15.82	0.030	0.045 *
Lower body (L)	41.33	40.67	0.849	0.878	9.32	30.03	*p* < 0.01	0.013 *	9.80	22.96	*p* < 0.01	0.014 *
Angulus (R, P)	64.54	63.88	0.844	0.878	23.68	40.97	*p* < 0.01	0.013 *	22.55	36.73	*p* < 0.01	0.014 *
Angulus (R, A)	48.98	50.24	0.720	0.878	24.18	38.68	*p* < 0.01	0.013 *	24.02	32.14	*p* < 0.01	0.014 *
Angulus (R, L)	21.17	20.10	0.705	0.878	7.05	14.25	*p* < 0.01	0.025 *	8.82	13.78	0.020	0.029 *
Antrum (G)	8.16	6.94	0.509	0.878	2.52	5.85	0.019	0.087	1.96	4.59	0.160	0.216
Antrum (P)	13.52	11.48	0.381	0.725	2.02	7.38	*p* < 0.01	0.013 *	2.45	6.12	0.070	0.102
Antrum (A)	8.67	10.77	0.316	0.725	1.76	8.4	*p* < 0.01	0.013 *	2.45	7.14	*p* < 0.01	0.019 *
Antrum (L)	13.27	13.40	0.956	0.956	2.52	10.18	*p* < 0.01	0.013 *	1.96	7.14	*p* < 0.01	0.019 *
Duodenal bulb	0.00	0.00	1.000	1	0.00	0.00	1.000	1	0.00	0.00	1.000	1
Duodenal descending	0.00	0.00	1.000	1	0.00	0.00	1.000	1	0.00	0.00	1.000	1

* *p*-values adjusted for multiple comparisons using the Benjamini–Hochberg false discovery rate (FDR) procedure. *p* < 0.05 indicates statistical significance.

**Table 3:** Comparison of missed diagnosis rates for various lesions between the EndoAdd training group and the traditional training group.

Lesion Type	Practice Phase (AI Detect/Actual/%)	Practice Phase (Traditional Detect/Actual/%)	*p*-Value	FDR	Testing Phase (AI Detect/Actual/%)	Testing Phase (Traditional Detect/Actual/%)	*p*-Value	FDR
SESCC	4/6 (66.7%)	2/6 (33.3%)	0.500	1.000	1/1 (100%)	1/1 (100%)	1.000	1.000
H. pylori infection	162/170 (95.3%)	166/178 (93.3%)	0.747	1.000	43/45 (95.6%)	41/43 (95.3%)	0.674	1.000
Esophageal SMT	20/20 (100%)	16/18 (88.9%)	0.470	1.000	3/3 (100%)	4/4 (100%)	1.000	1.000
EGC	16/18 (88.9%)	14/20 (70.0%)	0.333	1.000	4/5 (80.0%)	3/5 (60.0%)	0.500	1.000
Gastric polyp	76/76 (100%)	66/66 (100%)	1.000	1.000	13/13 (100%)	15/15 (100%)	1.000	1.000
Gastric ulcer	48/48 (100%)	52/52 (100%)	1.000	1.000	12/12 (100%)	11/11 (100%)	1.000	1.000
Gastric SMT	8/10 (80.0%)	8/10 (80.0%)	1.000	1.000	2/2 (100%)	2/2 (100%)	1.000	1.000
Duodenum/descending ulcer	36/36 (100%)	32/32 (100%)	1.000	1.000	11/11 (100%)	7/7 (100%)	1.000	1.000
Duodenum/descending SMT	4/4 (100%)	2/2 (100%)	1.000	1.000	0/0 (NA)	1/1 (100%)	NA	NA
Other	22/24 (91.7%)	28/32 (87.5%)	0.611	1.000	7/8 (87.5%)	8/10 (80.0%)	0.588	1.000

*p*-values adjusted for multiple comparisons using the Benjamini–Hochberg false discovery rate (FDR) procedure. *p* < 0.05 indicates statistical significance. SESCC, superficial esophageal squamous cell carcinoma; SMT, submucosal tumor; EGC, early gastric cancer.

## Data Availability

The EndoAdd system was developed in-house using PyTorch and is currently deployed in a research environment for validation purposes. To promote transparency and facilitate replication, the source code and trained model (excluding patient data) are available for non-commercial research use upon reasonable request to the corresponding author. Researchers interested in accessing the system or replicating the study may contact shuowang@fudan.edu.cn to initiate a data/materials transfer agreement.

## Supplementary Materials

The following supporting information can be downloaded at: https://www.mdpi.com/article/10.3390/bioengineering12060582/s1, Supplementary Table S1: Model performance on the classification of EGD images. AUC is the area under the receiver operating characteristics curve calculated on the test set; Supplementary Table S2: Comparison of pre- and post-training results for the EndoAdd trained endoscopists (Doctor I–IV); Supplementary Table S3: Comparison of pre- and post-training results for the traditional trained endoscopists (Doctor V–VIII).
