# Supplementary material for: Expert-AI Collaborative Training for Novice Endoscopists: A Path to Enhanced Efficiency
Source: Bioengineering (Basel). 2025 May 28;12(6):582. doi: 10.3390/bioengineering12060582 (PMC12189645; doi:10.3390/bioengineering12060582)
Supplement: Supplementary file 1 [file bioengineering-12-00582-s001.zip › bioengineering-3547922-supplementary.pdf]

**Supplementary Table S1: Model performance on the classification of EGD images. AUC is area under the receiver operating characteristics curve calculated on the test set.**

| Region | AUC | Region | AUC |
|---|---|---|---|
| **Esophage** | 0.995 | **Lower body (G)** | 0.944 |
| **Gastroesophageal junction** | 0.986 | **Lower body (P)** | 0.982 |
| **Fundus (G)** | 0.991 | **Lower body (A)** | 0.968 |
| **Fundus (P)** | 0.988 | **Lower body (L)** | 0.979 |
| **Fundus (A)** | 0.990 | **Angulus (R, P)** | 0.996 |
| **Fundus (L)** | 0.993 | **Angulus (R, A)** | 0.994 |
| **Middle-upper (G)** | 0.991 | **Angulus (R, L)** | 0.993 |
| **Middle-upper (P)** | 0.985 | **Antrum (G)** | 0.988 |
| **Middle-upper (A)** | 0.973 | **Antrum (P)** | 0.981 |
| **Middle-upper (L)** | 0.979 | **Antrum (A)** | 0.969 |
| **Middle-upper body (R, P)** | 0.967 | **Antrum (L)** | 0.991 |
| **Middle-upper body (R, A)** | 0.969 | **Duodenal bulb** | 0.999 |
| **Middle-upper body (R, L)** | 0.989 | **Duodenal descending** | 1.000 |
| **Mean AUC** | | 0.984 | |
| **Overall Accuracy** | | 98.0% | |

## Supplementary Table S2: Comparison of pre- and post-training results for the EndoAdd trained endoscopists (Doctor No.1-No.4)

| | Dr No.1 | | | Dr No.2 | | | Dr No.3 | | | Dr No.4 | | |
|---|---|---|---|---|---|---|---|---|---|---|---|---|
| | Practice n=192 | Testing n=48 | p | Practice n=204 | Testing n=53 | p | Practice n=209 | Testing n=53 | p | Practice n=184 | Testing n=50 | p |
| **Training Phase Score （30-point scale）** | 27 | | | 28 | | | 24 | | | 23 | | |
| **Inspection time Mean±SD)** | 6.33±1.36 | 5.85±1.41 | **0.032** | 6.30±1.45 | 5.79±1.61 | **0.028** | 6.23±1.45 | 5.62±1.65 | **0.016** | 6.36±1.51 | 5.80±1.37 | **0.013** |
| **Blind Spot n, range)** | 4.51±2.95 | 2.08±3.10 | **<0.01** | 4.37±2.69 | 2.100±2.93 | **<0.01** | 4.07±2.96 | 2.36±3.187 | **<0.01** | 3.35±2.48 | 1.54±1.88 | **<0.01** |
| **Photo documentation completeness (n%, range)** | 56 (22, 84) | 71 (29, 91) | **<0.01** | 56 (23, 84) | 74 (18, 92) | **<0.01** | 58 (13, 84) | 72 (20, 90) | **<0.01** | 60 (31, 88) | 76 (36, 92) | **<0.01** |
| **Biopsy** | 33.85 | 52.08 | **0.020** | 30.39 | 47.17 | **0.020** | 38.76 | 49.69 | 0.170 | 35.87 | 48.00 | 0.120 |
| **Esophage** | 0.00 | 0.00 | 1.000 | 0.00 | 0.00 | 1.000 | 0.00 | 0.00 | 1.000 | 0.00 | 0.00 | 1.000 |
| **Gastroesophageal junction** | 1.04 | 0.00 | 0.480 | 0.00 | 1.89 | 0.050 | 0.48 | 0.00 | 0.610 | 0.00 | 0.00 | 1.000 |
| **Fundus (G)** | 3.65 | 0.00 | 0.180 | 5.39 | 1.89 | 0.280 | 4.78 | 1.89 | 0.350 | 2.72 | 0.00 | 0.240 |
| **Fundus (P)** | 16.67 | 2.08 | **0.010** | 10.29 | 3.77 | 0.140 | 8.61 | 5.66 | 0.480 | 7.07 | 10.00 | 0.490 |
| **Fundus (A)** | 6.77 | 0.00 | 0.060 | 6.86 | 0.00 | 0.050 | 5.26 | 0.00 | 0.090 | 5.98 | 6.00 | 1.000 |
| **Fundus (L)** | 9.38 | 4.17 | 0.240 | 7.84 | 0.00 | **0.040** | 2.39 | 3.77 | 0.580 | 10.33 | 8.00 | 0.620 |
| **Middle-upper (G)** | 9.38 | 4.17 | 0.240 | 14.71 | 3.77 | **0.030** | 5.26 | 1.89 | 0.290 | 10.33 | 2.00 | 0.060 |
| **Middle-upper (P)** | 31.77 | 25.00 | 0.360 | 31.86 | 15.09 | **0.020** | 30.14 | 20.75 | 0.180 | 26.63 | 16.00 | 0.120 |
| **Middle-upper (A)** | 41.15 | 16.67 | **<0.01** | 40.69 | 16.98 | **<0.01** | 33.01 | 20.75 | 0.080 | 25.54 | 2.00 | **<0.01** |
| **Middle-upper (L)** | 30.21 | 8.33 | **<0.01** | 32.35 | 15.09 | **0.010** | 38.28 | 15.09 | **<0.01** | 31.52 | 12.00 | **0.010** |
| **Middle-upper body (R, P)** | 30.21 | 10.42 | **0.010** | 25.00 | 9.43 | **0.010** | 17.70 | 13.21 | 0.440 | 5.98 | 10.00 | 0.320 |
| **Middle-upper body (R, A)** | 35.42 | 14.58 | **0.010** | 30.88 | 18.87 | 0.080 | 29.19 | 24.53 | 0.500 | 24.46 | 10.00 | **0.030** |
| **Middle-upper body (R, L)** | 23.44 | 12.50 | 0.100 | 23.53 | 9.43 | **0.020** | 21.53 | 9.43 | 0.050 | 16.85 | 4.00 | **0.020** |
| **Lower body (G)** | 21.88 | 12.50 | 0.150 | 20.59 | 7.55 | **0.030** | 19.14 | 11.32 | 0.180 | 21.20 | 8.00 | **0.030** |
| **Lower body (P)** | 16.15 | 10.42 | 0.320 | 18.63 | 11.32 | 0.210 | 17.70 | 11.32 | 0.260 | 17.39 | 6.00 | 0.050 |
| **Lower body (A)** | 20.31 | 8.33 | 0.050 | 16.18 | 9.43 | 0.220 | 18.18 | 11.32 | 0.230 | 8.70 | 4.00 | 0.270 |
| **Lower body (L)** | 24.48 | 8.33 | **0.010** | 24.51 | 13.21 | 0.080 | 27.75 | 13.21 | **0.030** | 23.91 | 4.00 | **<0.01** |
| **Angulus (R, P)** | 51.56 | 25.00 | **<0.01** | 44.12 | 20.75 | **<0.01** | 44.98 | 26.42 | **0.010** | 34.78 | 18.00 | **0.020** |
| **Angulus (R, A)** | 36.98 | 27.08 | 0.200 | 38.24 | 24.53 | 0.060 | 38.28 | 20.75 | **0.020** | 32.07 | 24.00 | 0.270 |
| **Angulus (R, L)** | 14.58 | 8.33 | 0.260 | 16.18 | 3.77 | **0.020** | 14.83 | 15.09 | 0.960 | 10.33 | 8.00 | 0.620 |
| **Antrum (G)** | 8.33 | 4.17 | 0.330 | 5.88 | 1.89 | 0.240 | 4.78 | 1.89 | 0.350 | 2.17 | 0.00 | 0.290 |
| **Antrum (P)** | 6.77 | 2.08 | 0.220 | 8.33 | 5.66 | 0.520 | 8.13 | 1.89 | 0.110 | 7.61 | 0.00 | **0.040** |
| **Antrum (A)** | 5.73 | 2.08 | 0.300 | 5.39 | 1.89 | 0.280 | 7.18 | 3.77 | 0.370 | 2.17 | 2.00 | 0.940 |
| **Antrum (L)** | 5.21 | 2.08 | 0.360 | 9.31 | 3.77 | 0.190 | 9.57 | 1.89 | 0.070 | 7.07 | 0.00 | 0.050 |
| **Duodenal bulb** | 0.00 | 0.00 | 1.000 | 0.00 | 0.00 | 1.000 | 0.00 | 0.00 | 1.000 | 0.00 | 0.00 | 1.000 |
| **Duodenal descending** | 0.00 | 0.00 | 1.000 | 0.00 | 0.00 | 1.000 | 0.00 | 0.00 | 1.000 | 0.00 | 0.00 | 1.000 |

## Supplementary Table S3: Comparison of pre- and post-training results for the traditional trained endoscopists (Doctor No.5-No.8)

| | **Dr No.5** | | | **Dr No.6** | | | **Dr No.7** | | | **Dr No.8** | | |
|---|---|---|---|---|---|---|---|---|---|---|---|---|
| | **Practice n=215** | **Testing n=58** | **p** | **Practice n=192** | **Testing n=44** | **p** | **Practice n=205** | **Testing n=49** | **p** | **Practice n=199** | **Testing n=45** | **p** |
| **Training Phase Score （30-point scale）** | 29 | | | 26 | | | 25 | | | 24 | | |
| **Inspection time Mean±SD)** | 6.31±1.40 | 6.16±1.57 | 0.481 | 6.42±1.47 | 5.77±1.41 | **0.09** | 6.40±1.54 | 5.76±1.60 | 0.14 | 6.43±1.48 | 5.67±1.45 | **<0.01** |
| **Blind Spot n, range)** | 5.45±2.35 | 3.90±2.63 | **<0.01** | 5.23±2.09 | 3.32±1.90 | **<0.01** | 5.11±2.31 | 3.06±1.99 | **<0.01** | 4.78±2.20 | 3.56±2.49 | **<0.01** |
| **Photo documentation completeness (n%, range)** | 52 (22, 86) | 57 (28, 75) | **0.010** | 54 (18, 84) | 60 (35, 78) | **<0.01** | 52 (18, 79) | 60 (40, 70) | **<0.01** | 53 (25, 81) | 57 (30, 82) | **0.010** |
| **Biopsy** | 27.90 | 36.20 | 0.220 | 26.56 | 36.36 | 0.190 | 28.78 | 34.69 | 0.420 | 23.11 | 26.67 | 0.610 |
| **Esophage** | 0.00 | 0.00 | 1.000 | 0.00 | 0.00 | 1.000 | 0.00 | 0.00 | 1.000 | 0.00 | 0.00 | 1.000 |
| **Gastroesophageal junction** | 1.40 | 0.00 | 0.370 | 0.00 | 0.00 | 1.000 | 0.49 | 0.00 | 0.620 | 0.00 | 0.00 | 1.000 |
| **Fundus (G)** | 9.30 | 6.90 | 0.570 | 7.81 | 4.55 | 0.450 | 10.24 | 8.16 | 0.660 | 0.50 | 2.22 | 0.833 |
| **Fundus (P)** | 10.23 | 10.34 | 0.980 | 13.02 | 15.91 | 0.610 | 16.10 | 10.20 | 0.300 | 11.56 | 11.11 | 0.930 |
| **Fundus (A)** | 13.02 | 8.62 | 0.360 | 12.50 | 13.64 | 0.840 | 10.24 | 2.04 | 0.070 | 3.52 | 2.22 | 0.660 |
| **Fundus (L)** | 11.16 | 5.17 | 0.180 | 14.06 | 6.82 | 0.190 | 8.78 | 6.12 | 0.540 | 7.04 | 4.44 | 0.530 |
| **Middle-upper (G)** | 15.81 | 8.62 | 0.170 | 11.46 | 20.45 | 0.110 | 19.51 | 6.12 | **0.030** | 11.06 | 4.44 | 0.180 |
| **Middle-upper (P)** | 39.07 | 27.59 | 0.110 | 34.38 | 27.27 | 0.370 | 39.51 | 22.45 | **0.030** | 36.18 | 13.33 | **<0.01** |
| **Middle-upper (A)** | 49.77 | 25.86 | **<0.01** | 44.27 | 27.27 | **0.040** | 49.27 | 24.49 | **<0.01** | 41.21 | 24.44 | **0.040** |
| **Middle-upper (L)** | 38.60 | 15.52 | **<0.01** | 36.46 | 15.91 | **0.010** | 37.07 | 18.37 | **0.010** | 46.23 | 20.00 | **<0.01** |
| **Middle-upper body (R, P)** | 27.91 | 20.69 | 0.270 | 30.73 | 9.09 | **<0.01** | 17.07 | 14.29 | 0.640 | 18.59 | 24.44 | 0.370 |
| **Middle-upper body (R, A)** | 31.63 | 22.41 | 0.170 | 25.00 | 25.00 | 1.000 | 33.17 | 12.24 | **<0.01** | 32.16 | 28.89 | 0.670 |
| **Middle-upper body (R, L)** | 28.37 | 18.97 | 0.150 | 28.13 | 25.00 | 0.680 | 31.71 | 20.41 | 0.120 | 30.15 | 20.00 | 0.170 |
| **Lower body (G)** | 26.98 | 24.14 | 0.660 | 27.60 | 13.64 | 0.050 | 23.90 | 20.41 | 0.600 | 22.61 | 17.78 | 0.480 |
| **Lower body (P)** | 23.26 | 22.41 | 0.890 | 23.44 | 9.09 | **0.030** | 18.05 | 12.24 | 0.330 | 21.61 | 17.78 | 0.570 |
| **Lower body (A)** | 18.60 | 17.24 | 0.810 | 20.83 | 6.82 | **0.030** | 21.95 | 18.37 | 0.580 | 12.56 | 20.00 | 0.190 |
| **Lower body (L)** | 39.53 | 27.59 | 0.090 | 34.38 | 22.73 | 0.140 | 31.71 | 16.33 | **0.030** | 36.18 | 24.44 | 0.130 |
| **Angulus (R, P)** | 48.37 | 44.83 | 0.630 | 48.96 | 31.82 | **0.040** | 57.07 | 24.49 | **<0.01** | 56.78 | 44.44 | 0.130 |
| **Angulus (R, A)** | 47.44 | 31.03 | **0.030** | 48.96 | 36.36 | 0.130 | 40.49 | 26.53 | 0.070 | 41.71 | 35.56 | 0.450 |
| **Angulus (R, L)** | 16.74 | 13.79 | 0.590 | 17.19 | 13.64 | 0.570 | 16.10 | 18.37 | 0.700 | 19.10 | 8.89 | 0.100 |
| **Antrum (G)** | 10.70 | 6.90 | 0.390 | 7.29 | 2.27 | 0.220 | 2.93 | 4.08 | 0.680 | 4.52 | 4.44 | 0.980 |
| **Antrum (P)** | 11.16 | 10.34 | 0.860 | 11.98 | 2.27 | 0.060 | 7.32 | 6.12 | 0.770 | 7.54 | 4.44 | 0.460 |
| **Antrum (A)** | 9.30 | 12.07 | 0.530 | 11.98 | 2.27 | 0.060 | 9.27 | 6.12 | 0.480 | 8.04 | 6.67 | 0.760 |
| **Antrum (L)** | 16.28 | 8.62 | 0.140 | 13.02 | 0.00 | **0.010** | 8.78 | 8.16 | 0.890 | 9.05 | 11.11 | 0.670 |
| **Duodenal bulb** | 0.00 | 0.00 | 1.000 | 0.00 | 0.00 | 1.000 | 0.00 | 0.00 | 1.000 | 0.00 | 0.00 | 1.000 |
| **Duodenal descending** | 0.00 | 0.00 | 1.000 | 0.00 | 0.00 | 1.000 | 0.00 | 0.00 | 1.000 | 0.00 | 0.00 | 1.000 |
